# P-778. Evaluation of Fluoroquinolone Use for Pseudomonal Urinary Tract Infections

**DOI:** 10.1093/ofid/ofaf695.989

**Published:** 2026-01-11

**Authors:** Ludmila Nicov, Madiha Shah, Shu Lu, Kendra Salangsang, Hien Nguyen

**Affiliations:** Veterans Affairs Northern California Healthcare System, Antelope, CA; VA Northern California Health Care System, Mather, California; VA Northern California Health Care, Folsom, California; Veterans Affairs Northern California Healthcare System, Antelope, CA; VA Northern California Health Care System / UC Davis Health, Mather, California

## Abstract

**Background:**

Pseudomonas aeruginosa (PsAr) is responsible for roughly 10% of catheter-associated UTIs (CAUTIs) and up to 16% of ICU infections, often leading to complications such as bacteremia. Fluoroquinolones (FQs) remain one of the few oral treatment options for PsAr UTIs. Despite rising resistance, FQs like ciprofloxacin and levofloxacin achieve high urinary concentrations—potentially overcoming higher MICs and maintaining efficacy. In 2019, the CLSI lowered PsAr FQ breakpoints, raising questions about continued clinical effectiveness for higher MICs. This study aims to evaluate the use of FQs for treating pseudomonal UTIs and to assess clinical success in relation to MIC levels.

**Table 1.**
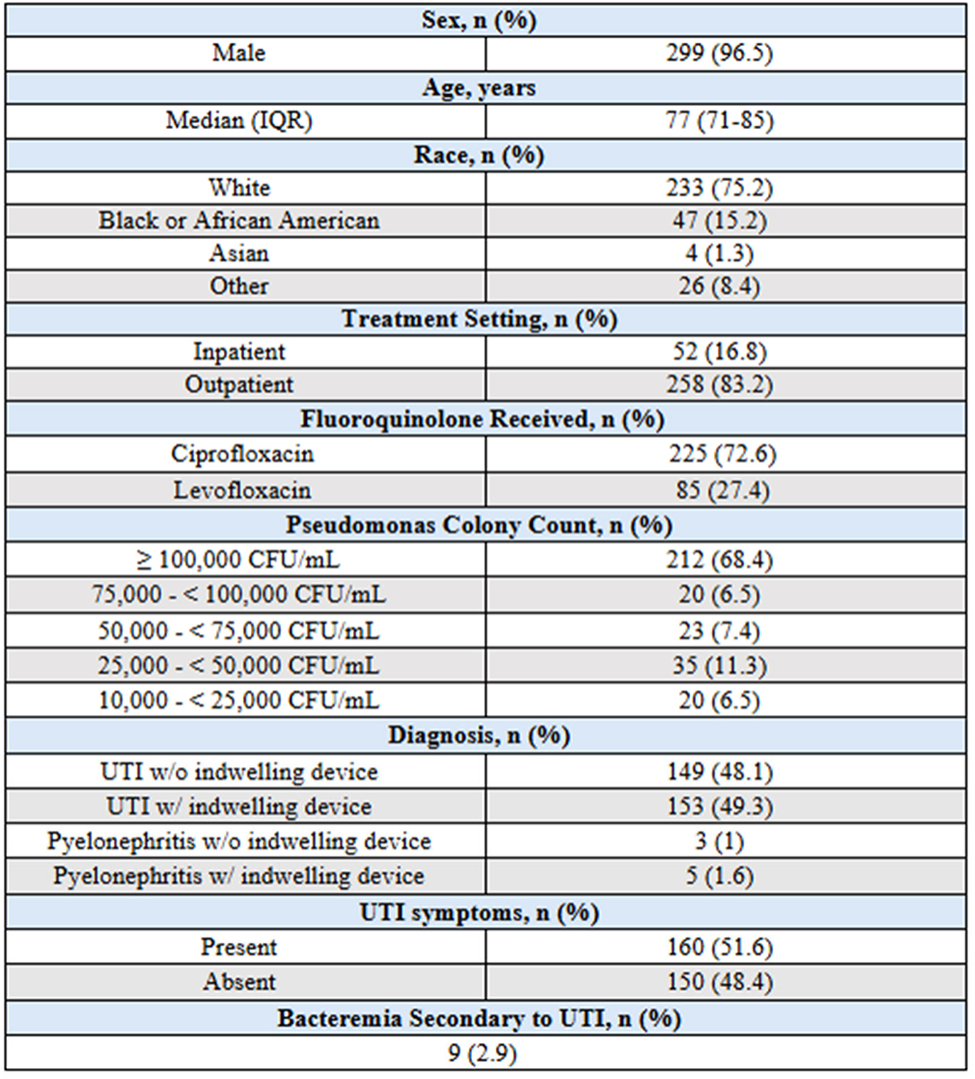
Baseline Characteristics

**Tables 2 & 3.**
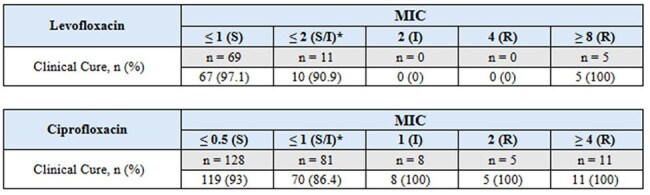
Primary Endpoint * Further breakdown of MIC not available

**Methods:**

This retrospective observational study analyzed adult patients treated with FQ for a PsAr UTI between January 1, 2017, and December 31, 2022. Inclusion criteria were a positive urine culture for PsAr and treatment with ciprofloxacin or levofloxacin. Patients with prostatitis were excluded. The primary outcome was clinical cure within 7 days post-therapy. Secondary outcomes included relapse or reinfection within 30 days, with evaluation of risk factors such as indwelling urinary devices and history of kidney stones.

**Tables 4 & 5.**
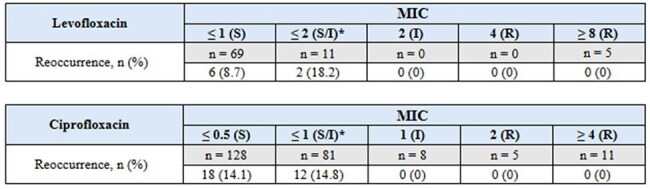
Secondary Endpoint * Further breakdown of MIC not available

**Results:**

Of 310 patients, 85 (27.4%) received levofloxacin and 225 (72.6%) received ciprofloxacin. Clinical cure within 7 days was achieved in 82 (96.5%) of the levofloxacin group and 205 (91%) of the ciprofloxacin group. All patients resistant to levofloxacin (n=5) or intermediate/resistant to ciprofloxacin (n=24) achieved 100% clinical cure. Recurrence within 30 days occurred in 8 (9%) of levofloxacin and 30 (12%) of ciprofloxacin patients; all recurrent cases remained susceptible to their respective fluoroquinolone. Among those with recurrence, 5 (62.5%) in the levofloxacin group and 18 (60%) in the ciprofloxacin group had indwelling catheters. No patients had a documented history of kidney stones by provider notes or imaging.

**Conclusion:**

FQs remain a primary oral treatment option for pseudomonal UTIs and may achieve clinical cure despite higher MICs. Further research is needed to determine if high urinary FQ concentrations improve outcomes against intermediate or resistant PsAr isolates.

**Disclosures:**

All Authors: No reported disclosures

